# Synthetic Biology-derived triterpenes as efficacious immunomodulating adjuvants

**DOI:** 10.1038/s41598-020-73868-6

**Published:** 2020-10-13

**Authors:** Mizuki Tateno, Barbara J. Stone, Sarah J. Srodulski, Stephanie Reedy, Thomas R. Gawriluk, Thomas M. Chambers, Jerold Woodward, Joe Chappell, Chase F. Kempinski

**Affiliations:** 1 Department of Pharmaceutical Sciences, University of Kentucky, Lexington, KY 40536-0596 USA; 2 ParaTechs Corporation, Lexington, KY 40505 USA; 3 Gluck Equine Research Center, University of Kentucky, Lexington, 40546-0099 USA; 4 Enepret Incorporated, Lexington, KY 40506 USA; 5 Department of Microbiology, Immunology and Molecular Genetics, University of Kentucky, Lexington, KY 40536-0298 USA

**Keywords:** Immunology, Vaccines, Adjuvants, Vaccines, Biotechnology

## Abstract

The triterpene oil squalene is an essential component of nanoemulsion vaccine adjuvants. It is most notably in the MF59 adjuvant, a component in some seasonal influenza vaccines, in stockpiled, emulsion-based adjuvanted pandemic influenza vaccines, and with demonstrated efficacy for vaccines to other pandemic viruses, such as SARS-CoV-2. Squalene has historically been harvested from shark liver oil, which is undesirable for a variety of reasons. In this study, we have demonstrated the use of a Synthetic Biology (yeast) production platform to generate squalene and novel triterpene oils, all of which are equally as efficacious as vaccine adjuvants based on physiochemical properties and immunomodulating activities in a mouse model. These Synthetic Biology adjuvants also elicited similar IgG1, IgG2a, and total IgG levels compared to marine and commercial controls when formulated with common quadrivalent influenza antigens. Injection site morphology and serum cytokine levels did not suggest any reactogenic effects of the yeast-derived squalene or novel triterpenes, suggesting their safety in adjuvant formulations. These results support the advantages of yeast produced triterpene oils to include completely controlled growth conditions, just-in-time and scalable production, and the capacity to produce novel triterpenes beyond squalene.

## Introduction

Early evidence of primitive vaccinations to prevent infectious ailments, such as smallpox, has been traced back to the tenth century^1^. Although the medical community of that time had no knowledge of adaptive immunity, we now know how crucial vaccinations are to protect the public from devastating and contagious diseases. While vaccination programs have been effective in nearly eliminating the presence of some diseases, many diseases remain a burden due to poor immunogenic responses generated by current immunizations or evolving pathogens. Perhaps the most familiar disease in the latter category is the influenza virus. Despite intense study and mechanistic understanding, influenza remains a constant presence and threat to much of the world. Between the Northern and Southern hemispheres, seasonal influenza exists year-round forcing the World Health Organization to create two separate influenza vaccines campaigns annually to cover both hemispheres^2,3^.

Seasonal influenza vaccines are crucial for the well-being of the public. However, two major sectors of the population are more vulnerable—the immunologically naïve (infants) and immunosenescent populations, primarily the elderly. Two common, but imperfect vaccination options are available for these groups: receive a higher antigen dose (immunosenescent) or receive two vaccinations over a short period of time (immunologically naïve). A third, more convenient and effective option is to receive an adjuvanted vaccine that significantly increases the antibody response. An adjuvant is a component in the vaccine formulation that improves the body’s response to the co-delivered antigen, enhancing the immune response to the target when compared to inoculation with antigen alone (although this may not completely eliminate the need for a boost immunization). Goodwin et al.^4^ published a review of 31 studies examining the antibody responses generated by influenza vaccines in young and elderly adult groups and found that the elderly had a lower immune response upon receiving the vaccine, suggesting that a more immunogenic vaccine is required. Oil-in-water (O/W) adjuvant systems have been investigated since the 1980s for their reduced toxicity (compared to water-in-oil adjuvants) to deliver emulsified muramyl dipeptide^5,6^, the minimal component needed to stimulate the immune system using whole killed mycobacteria^7^. Continued investigation into the efficacy of oil-in-water emulsions led to the development of the MF59 adjuvant in the mid-1990s by Chiron^8,9^, which demonstrated that the emulsion alone exhibited excellent adjuvant properties. Currently, the MF59-adjuvanted influenza vaccine has been approved by the Food and Drug Administration (FDA) and widely administered under the tradename FLUAD (Seqirus) since 2015 for people over the age of 65 in the United States. Recently, studies comparing MF59 adjuvanted and non-adjuvanted influenza vaccines in young infants have reported that the use of the MF59 adjuvant enhanced immunogenicity by inducing higher antibody titers and longer duration responses, leading to the use of an adjuvanted influenza vaccine for children in Canada^10–13^. The MF59 adjuvant’s utility is not solely for the purpose of the seasonal influenza vaccine. Studies investigating the efficacy of MF59 for pandemic influenza vaccines have also demonstrated the ability of MF59 to reach and satisfy the European Union’s CHMP criteria for vaccine licensure and allow for the induction of cross reactive, long lasting memory B cells^14,15^. MF59 also has demonstrated that it enhances cross reactive neutralizing antibody response to the pandemic influenza virus^16^. Furthermore, MF59 has been found to elicit a strong response across a variety of antigens such as for tuberculosis and chlamydia vaccine preparations^17^. Such findings suggest good potential for application over a wide variety of vaccines.

At its working concentration, MF59 is composed of 2.5% (v/v) squalene, 0.25% (w/v) Tween-80, and 0.25% (w/v) Span-85 emulsified in a 10 mM citrate buffer (pH 6.0) with a final droplet size of ~ 165 nm for review see^18^. This adjuvant creates an immunocompetent environment at the injection site by increasing antigen uptake and recruiting a greater number of monocytes and neutrophils^19^. The oil in MF59 is the triterpene squalene. Squalene was initially characterized from shark liver oil by Tsujimoto in 1916^20^, and the richest natural source of squalene still continues to be from deep-sea shark livers. Unfortunately, due to overfishing and increased pollution in the ocean (such as heavy metals and persistent organic pollutants) that can contaminate the triterpene product, shark-derived squalene is an unfavorable source for medical applications^21,22^. Crude olive (*Olea europa*) oil has been an alternative source of non-animal squalene for when purity requirements have been lessened, as in personal care applications. Olive oil is ~ 0.15% squalene by weight, with the triterpene being more easily obtained through the deodorized distillate—a byproduct in the olive oil refining infrastructure. However, because of the low amount of squalene and the complex chemical mixtures inherent to plant extracts, the final purity of olive-derived squalene is often ~ 97.5%, compared to the > 99% purity from the marine source^22^. While it is possible to obtain higher levels of purity from the plant source, it is not economical, and this discrepancy is critical in a demanding application like a vaccine. Despite this caveat, plant-derived squalene has been evaluated for its application in an emulsion adjuvant in *Neisseria meningitidis B* and influenza vaccines^23^. That investigation concluded that there was no difference in the antibody titers elicited between animal- and plant-sourced squalene.

**Figure 1:**
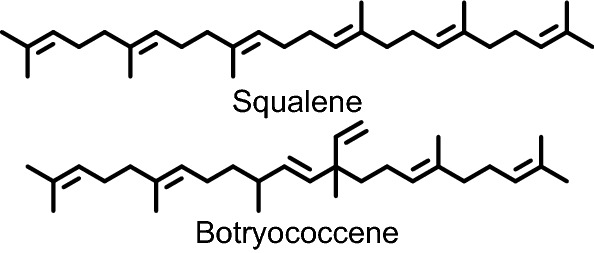
Chemical structure for botryococcene and squalene.

The development of biotechnological tools has started an era where limited natural products can be produced using Synthetic Biology via genetically engineered organisms, with the greatest successes using microbes for bioproduct manufacture^24^. We have developed a *Saccharomyces cerevisiae* expression platform^25^ that allows for the biosynthesis of squalene and similar triterpenes which addresses the needs for high purity and non-animal sourced triterpene oils. The design of the yeast chassis allows for the total biosynthetic production of squalene and other triterpene oils. More specifically, we are interested in applications for squalene isomers, such as botryococcene. Botryococcene is an isomer of squalene that is generated when the two precursor molecules, farnesyl diphosphate (FPP), are conjugated in an asymmetrical manner unlike squalene, giving rise to a more branched triterpene oil^26,27^ (Fig. 1). Botryococcene is naturally produced only by the green algae, *Botryococcus braunii*, which has specific races; one of which (race B) produces large amounts of triterpene oils, including methylated forms of both squalene and botryococcene^26,28^. While *B. braunii* can produce large amounts of triterpene oil (> 30% of its dry weight) its slow-growth habit^29^, resistance to extraction^30^, and mixture of chemically similar triterpene oils—which would require laborious purification to obtain large amounts of individually pure triterpenes—all make it a less desirable source. Yeast have several advantages such as ease of genetic modification, a large body of work in scaling bioreactor/fermentation processes, a prevalence of accessible infrastructure for scaling, and are a generally recognized as safe (GRAS) organism by the FDA^31^. The ability to easily genetically manipulate yeast also allows for more specific (or less complex) mixtures to be produced, allowing for easier purification.

The specific goals of this study were to compare the immune response among adjuvants composed of triterpenes produced using Synthetic Biology to the currently used marine sources in an emulsion adjuvant delivered with a commercially available quadrivalent seasonal influenza vaccine in a murine model. Pharmacology and toxicology research often use structure–activity relationships (SAR) to predict how a novel compound will behave by looking towards similar compounds and their activity. In a similar manner, we compared yeast-produced squalene, botryococcene, and a mixture of methylated squalenes as adjuvants in comparison to a commercially available emulsion (Addavax) and an in-house formulated shark squalene emulsion. Our goal was to compare the equivalency of Synthetic Biology squalene in adjuvant formulations to that derived from shark, as well as to perform the first of its kind SAR for structurally distinct triterpenes to elicit biologically significant increases in antibody and hemagglutination-inhibiting titers. Our results demonstrate the safety and efficacy of Synthetic Biology produced triterpene oils in emulsion adjuvant formulations.

## Methods

### Biosynthetic production and purification of triterpenes

The *S. cerevisiae* strains used were based on the ZX lines previously described^25^. Briefly, yeast were simultaneously selected for aerobic sterol auxotrophy, a constitutive gain-of-function *Sterol Uptake Enhancement* (*SUE*) mutation, and production of high amounts of the triterpene precursor, FPP. Squalene or botryococcene production was obtained through introduction of a full-length *B. braunii* squalene synthase gene (BSS) or the squalene synthase-like (SSL) fusion protein of *B. braunii* SSL-1 and SSL-3 that includes a membrane associating region, respectively^32^. The mixture of methylated (C31 and C32) and non-methylated (C30) squalenes were obtained by integrating a copy of BSS and a copy of *B. braunii* triterpene methyltransferase 1^26^ into the yeast genome.

Triterpenes were extracted from yeast cultures by adding acetone and hexane to the aqueous culture for a final ratio of 1:1:1 (acetone:hexane:aqueous). The mixture was shaken and allowed to separate into organic and aqueous phases. The upper organic phase was collected and condensed by rotoevaporation. The condensed extract was purified over a silica column using hexane as an eluent. The triterpene fraction was assessed for purity via gas chromatography–mass spectrometry and the singly purified triterpenes were also verified using nuclear magnetic resonance spectroscopy with comparison to analytical standards (Supplemental Fig. 1).

### Adjuvant and vaccine formulation

The nanoemulsions were produced as previously described^23^ and were measured for their physiochemical properties. A mixture of 5% triterpene oil (v/v), 0.5% Tween-80 (w/v), and 0.5% Span-85 (w/v) suspended in a 20 mM citrate buffer (pH 6.0) was passed through an Avestin EmulsiFlex-C5 high pressure homogenizer at 12,000 PSI at least eight times. The nanoemulsion was then passed through a 0.2 µm filter and the particle size was measured using a Malvern Nano Zetasizer Dynamic Light Scattering (DLS) spectrophotometer. Shark squalene was obtained from Sigma-Aldrich and Addavax (a squalene nanoemulsion commercially available adjuvant) from InvivoGen. The adjuvants were combined 1:1 with the quadrivalent influenza antigen that had been diluted with 2 × PBS to create the adjuvanted vaccine. The influenza antigen used was the 2018–2019 Afluria vaccine (Seqirus), a non-adjuvanted quadrivalent influenza vaccine (H1N1A, H3N2, B/Maryland/15/2016, and B/Phuket/3073/2013-BVR-1B). The antigen-only control was formulated by mixing 20 mM citrate buffer (pH 6.0) with an equal amount of Afluria antigen. The final concentration of total antigens in each vaccine was 0.36 µg (0.09 µg per individual antigen). The Pierce Chromogenic Endotoxin Quant kit (Thermo Fisher) was used for endotoxin detection.

### Animals

All applicable international, national, and institutional guidelines for the care and use of animals were followed. All procedures performed in studies involving animals were in accordance with the ethical standards of the Institutional Animal Care and Use Committees at ParaTechs Corporation (Lexington, KY, USA), where the studies were conducted. The ParaTechs Corporation IACUC committee approved the studies and ParaTechs Corporation performed all studies involving animals under an Assurance of Compliance filed with the Office of Laboratory Animal Welfare, National Institutes of Health, Public Health Service, United States Department of Health and Human Services. Animal housing and use was conducted in accordance with the Guide for the Care and Use of Laboratory Animals, 8th edition^33^. BALB/c (BALB/cAnNCrl) specific pathogen-free mice were obtained from Charles River Laboratories. Mice were 8 weeks old (~18 g) at the start of the experiment with equal number of male and female mice for each treatment (eight of each sex). The vivarium was maintained at 20–22 °C with an average relative humidity of 35–75% under a 12:12 h light:dark cycle. Mice were housed in standardized ventilated microisolation caging (Innovive). Animals were acclimated for a minimum of 7 days prior to treatment. Mice had access to Teklad Global 19% protein extruded rodent diet #2919 (Envigo) and water ad libitum.

### Immunizations and blood collections

**Figure 2:**
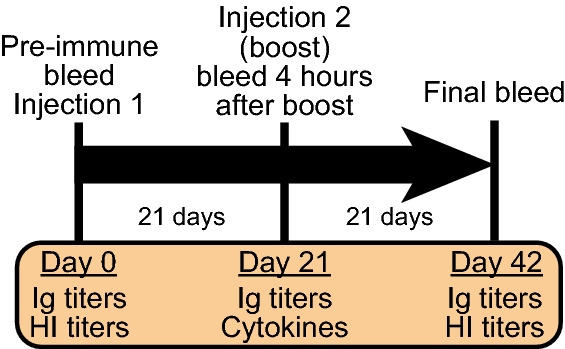
The experimental timeline for immunization, blood collection, and data analysis in this study.

In each immunization, mice received a total of 100 μL of vaccine, with 50 μL injected subcutaneously into each hind leg (shaved). Due to availability of certain adjuvant mixes, certain treatment groups were enlarged with an equal number of both sexes. The number of animals used in each treatment is presented in each figure legend. Mice were immunized twice, 21 days apart. Blood samples were taken three times: immediately prior to initial immunization (day 0, pre-immune); four hours after the second immunization (day 21, boost); and at the end of the study (day 42, final) (Fig. 2). Blood samples for analysis were collected on day 0 and day 21 via submandibular collection. Blood samples collected on day 42 were performed by cardiac puncture under terminal anesthesia using 3% inhaled isoflurane delivered with O_2_ using a VetEquip inhalation anesthesia system (Pleasanton). After euthanasia, mice were evaluated externally and internally for potential pathologic effects of immunizations including the injection site, spleen, liver, kidneys, and Peyer’s patches.

### ELISA (Total IgG, IgE, IgG2a, IgG1) and cytokine assay

Total IgG was quantified by ELISA (Mouse IgG total ELISA Ready-SET-Go! kit, eBioscience). Total IgG titers were assayed against the quadrivalent antigen. Titers of the individual antibodies were determined by coating 96 well plates (Nunc Maxisorp) with 0.08 µg quadrivalent antigen per well using a basic carbonate buffer and incubated overnight at 4 °C. The following day, the plates were washed three times with the wash buffer (1 × PBS with 0.05% Tween 20, pH 7.4) and blocked for an hour at room temperature with 100 µL per well of 1% bovine serum albumin (BSA) (Thermo Fisher). The plates were washed four times, the serum samples were serially diluted onto the blocked plates in duplicate and incubated at room temperature for one hour. After incubation and subsequent washing, a secondary antibody was added: goat anti-mouse IgG1, rat anti-mouse IgG2a, or goat anti-mouse IgE (Invitrogen), and the plates were incubated for an hour at room temperature. Pierce 3,3′,5,5′-tetramethylbenzidine (TMB) substrate solution (Thermo Fisher) was added to the washed plates, incubated at room temperature for 15 min, the reaction was stopped, and the plates’ absorbance at 450 nm was recorded. The greatest serum dilution that gave a positive reading, i.e. the titer absorbance that was greater than the blank’s (no serum) mean plus three times the blank’s standard deviation, was recorded as the immunoglobulin titer^23^. IgE antibodies against botryococcene were analyzed by ELISA as described by Matyas et al., 2002^34^ with modifications. Botryococcene (1 µL) was mixed into 10 mL of isopropyl alcohol and 100 µL of the mixture was used to coat the wells of a 96 well polystyrene plate. The plates were left overnight at room temperature in a chemical fume hood to allow the alcohol to evaporate. The following day, the plates were blocked at room temperature for two hours with 300 µL of a 1 × PBS with 2% BSA solution. Afterwards, the blocking solution was removed and 100 µL of the serum samples (1:50 dilution in 1 × PBS with 2% BSA) was added. The plate was incubated at room temperature for one hour, followed by four washes with 1 × PBS. Secondary antibody (goat anti-mouse IgE secondary antibodies from Invitrogen) was diluted in 1 × PBS with 2% BSA (1:1000) and 100 µL added to each well. The plate was again incubated for one hour at room temperature followed by four washes with 1 × PBS. Pierce TMB substrate solution (Thermo Fisher) was added to the washed plates, incubated at room temperature for 15 min, the reaction was stopped, and the plates’ absorbances at 450 nm were recorded.

Cytokine levels were analyzed in serum using the Q-Plex Mouse Cytokine inflammation (14 Plex) kit (Quansys) according to the manufacturer’s instruction. All samples were run in duplicate.

### Hemagglutination inhibition (HI) assay

HI titers were quantified from serum using the hemagglutination-inhibition assay as previously reported^35^. Non-specific inhibitors in serum samples were inactivated by treating the aliquots of each serum with receptor destroying enzyme (RDE) II from Denka Seiken, followed by a 30-min incubation at 56 °C prior to testing. Serum samples were serially diluted two-fold into U-bottom 96 well microtiter plates, starting at a 1:10 dilution and incubated with the H3N2 Influenza A virus (A/Singapore/INFIMH-16-0019/2016 (H3N2)) followed by the addition of adult turkey red blood cells. All sera were run in duplicate and the HI titer were defined as the last serum dilution in which the complete agglutination inhibition occurred. Influenza A Virus, A/Singapore/INFIMH-16-0019/2016 (H3N2), FR-1590, was obtained through the International Reagent Resource, Influenza Division, WHO Collaborating Center for Surveillance, Epidemiology and Control of Influenza, Centers for Disease Control and Prevention, Atlanta, GA, USA. Ferret antisera, used as positive control, to Influenza A Virus, A/Singapore/INFIMH-16-0019/2016 (H3N2), FR1618, was obtained through the International Reagent Resource, Influenza Division, WHO Collaborating Center for Surveillance, Epidemiology and Control of Influenza, Centers for Disease Control and Prevention, Atlanta, GA, USA.

### Data and statistical analysis

Data was analyzed using GraphPad Prism (version 8.3.0). Group values were compared using a Kruskal–Wallis test with Dunn’s multiple comparison test (α = 0.05).

## Results

### Yeast triterpene nanoemulsions exhibit equivalent physiochemical properties to current standards

Three separate Synthetic Biology-derived O/W adjuvants modeled after the composition of MF59 were created for the purpose of this study: C30 squalene, C30 botryococcene and mixed methylated (C30:C31:C32 [95:3:2]) squalenes formulations. An adjuvant using shark-derived squalene was also generated as an in-house formulation control. DLS spectroscopy indicated the average size of the emulsion droplets were 179.88 nm, 183.97 nm, 175.13 nm and 196.47 nm for the shark squalene, yeast derived squalene, botryococcene and mixed squalene, respectively (Table 1). The nanoemulsions that were produced using the yeast triterpenes were similar in size compared to the formulation produced using the shark squalene and to Addavax. The polydispersity index (PDI) was also measured for all samples and were similar to the control, Addavax (Table 1). In addition, antigen integrity was verified by SDS-PAGE^36^ and zinc staining after the adjuvants were formulated with the influenza vaccine and exhibited no difference compared to antigen only (Supplemental Fig. 2). Adjuvant stability was evaluated after storage at 4 °C for 24 months and measured again using DLS which indicated less than a 5 nm change in particle size of the adjuvants produced using the yeast-derived triterpenes (Supplemental Fig. 3).

**Table 1:** Nanoparticle size of adjuvants formulated with different triterpenes.

Adjuvant	Average particle size (nm) ± SD	Average PDI ± SD
AddaVax	170.90 ± 3.66	0.143 ± .024
Shark squalene	179.88 ± 1.59	0.135 ± .025
Yeast squalene	183.97 ± 2.68	0.134 ± .008
Yeast botryococcene	175.13 ± 1.97	0.139 ± .006
Yeast mixed squalene	196.47 ± 3.56	0.161 ± .008

Adjuvant formulations’ average particle sizes and polydispersity indices (PDI) as determined by DLS spectroscopy.

### Synthetic Biology squalene and novel triterpenes do not exhibit reactogenic effects

Vaccines were delivered subcutaneously in the shaven hindquarters of BALB/c mice. The vaccine site was kept shaved to monitor for any morphological reactogenic effects. Some mice exhibited a raised white area around the injection site, which appeared at varying times post-injection (days to weeks after immunization) but subsided within a few days. Beyond this, it did not appear to cause any discomfort and most likely would have gone unnoticed if the area had not been shaved. These phenomena appeared more in adjuvanted versus antigen only vaccines but did not consistently occur more in any specific adjuvant formulation. While we found few other reports of similar pathology in the literature, it was most similar to a result observed with the RIBI emulsion adjuvant^37^. None of the yeast-produced triterpene formulated adjuvants exhibited any reactogenic morphologies nor were there any consistent gross changes to the spleen, liver, kidneys, or Peyer’s patches at the end of the experiment in any of the treatments.

### Yeast-derived triterpene adjuvants match immunological profiles of marine and commercial adjuvants

**Figure 3:**
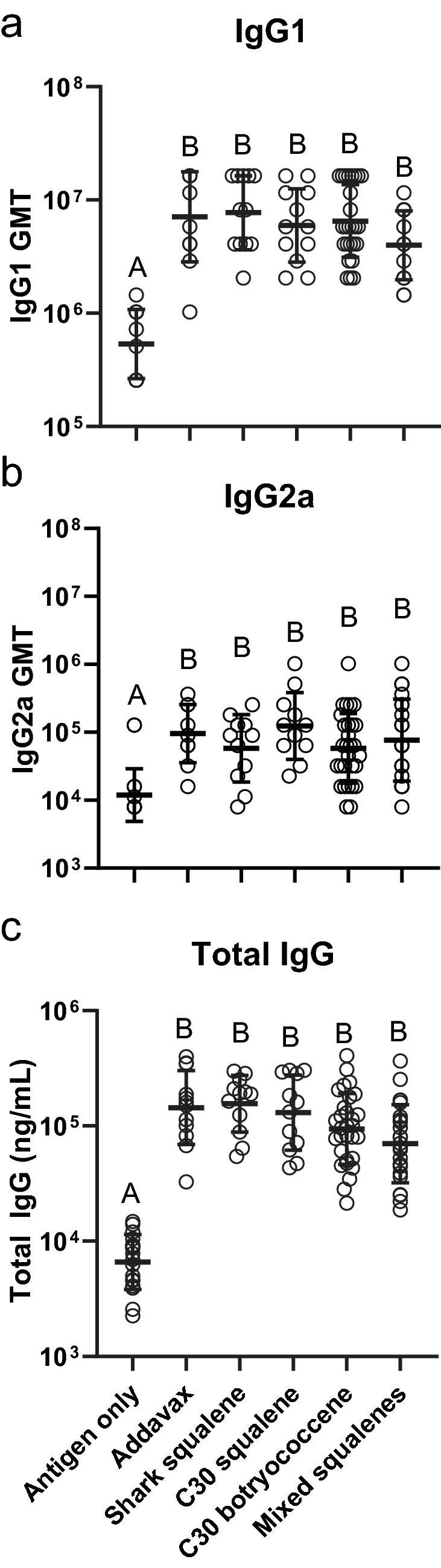
Antibody responses of mice to influenza vaccine formulated with different triterpene adjuvants. (**a**) IgG1, (**b**) IgG2A, and (**c**) Total IgG against quadrivalent antigen were quantified in serum using ELISA. Serum was taken on day 42. GMT are presented from samples determined in duplicate (± geometric standard deviation). Group values were compared using a Kruskal–Wallis test with Dunn’s multiple comparison test (α = 0.05). Different letters above groups represent significant differences, shared letters represent no significant differences. Open circles represent individual animals (males and females). Antigen only (IgG1: n = 16; Total IgG and IgG2a: n = 18), Addavax (n = 12), shark squalene (IgG1 and total: n = 12; IgG2a: n = 11), C30 squalene (IgG1 and total: n = 12; IgG2a: n = 11), C30 botryococcene (IgG1: n = 26; Total and IgG2a: n = 30), mixed squalenes (IgG1: n = 13; IgG2a: n = 21; Total: n = 24).

**Figure 4:**
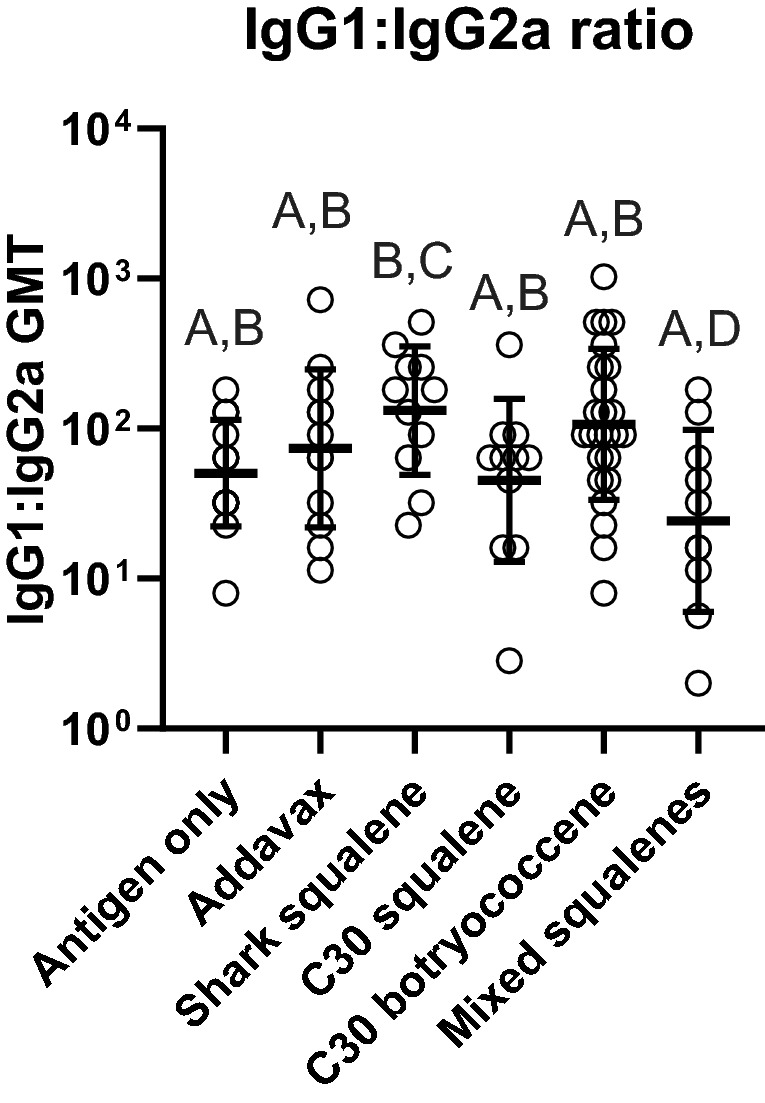
Comparison of antibody ratios as an indicator of Th1 versus Th2 responses. Ratios were calculated from the IgG1 and IgG2a determinations as shown in Fig. 3, the data used was for paired (IgG1 and IgG2) mice serum taken on day 42. Antigen only (IgG1: n = 16; Total IgG and IgG2a: n = 18), Addavax (n = 12), shark squalene (IgG1 and total: n = 12; IgG2a: n = 11), C30 squalene (IgG1 and total: n = 12; IgG2a: n = 11), C30 botryococcene (IgG1: n = 26; Total and IgG2a: n = 30), mixed squalenes (IgG1: n = 13; IgG2a: n = 21; Total: n = 24). GMT are presented from samples determined in duplicate (± geometric standard deviation). Group values were compared using a Kruskal–Wallis test with Dunn’s multiple comparison test (α = 0.05). Different letters above groups represent significant differences, shared letters represent no significant differences. Open circles represent individual animals (males and females).

**Figure 5:**
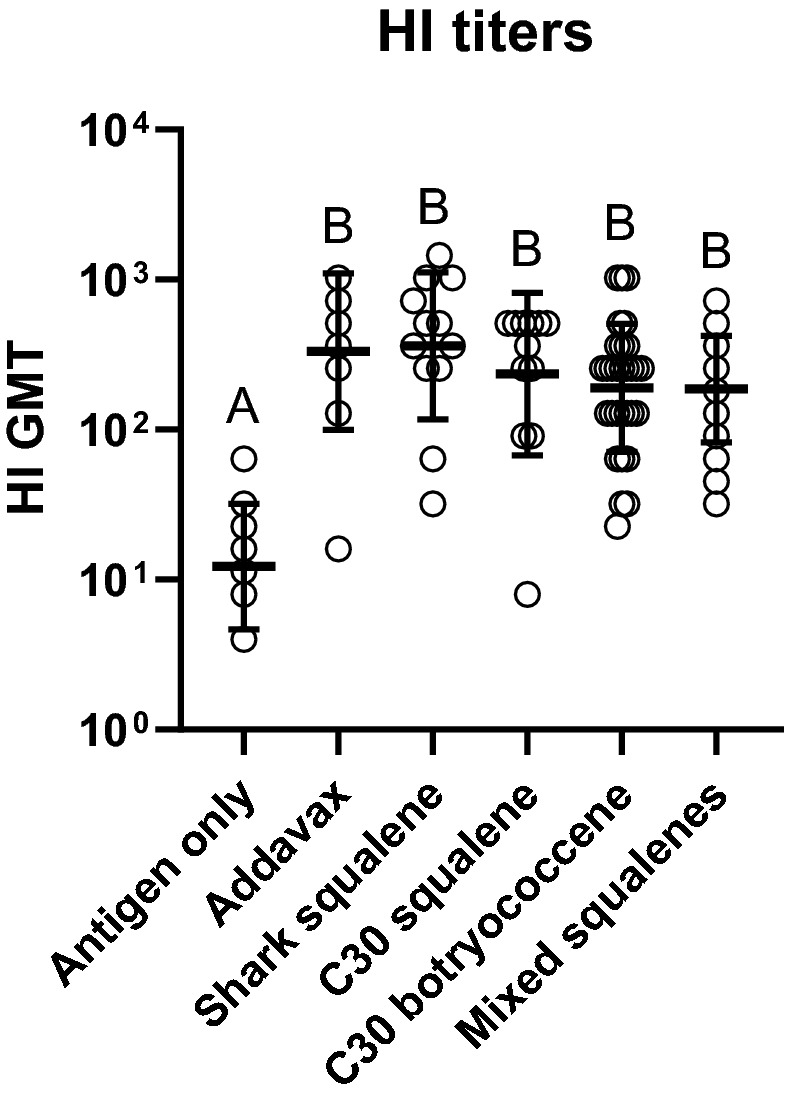
HI titers determined for influenza vaccine formulated with different triterpene adjuvants. Titers were quantified from serum using the RDE (II) "Seiken" protocol. Serum was taken on day 42. Pooled pre-inoculation serum showed no responses. GMT are presented from samples determined in duplicate (+/− geometric standard deviation). Group values were compared using a Kruskal–Wallis test with Dunn’s multiple comparison test (α = 0.05). Different letters above groups represent significant differences, shared letters represent no significant differences. Open circles represent individual animals. Antigen only (n = 18), Addavax (n = 12), shark squalene (n = 12), C30 squalene (n = 12), C30 botryococcene (n = 30), mixed squalenes (n = 24).

The Synthetic Biology triterpene adjuvants were evaluated for their ability to increase the serum immune response through ELISAs against the vaccine antigens. Serum immunoglobulins assayed were IgG1, IgG2a, IgE, and total IgG. Sera collected after the boost immunization (21 days) and at the end of study (42 days) were analyzed (Fig. 2). IgG1 and IgG2a, for the combined sexes showed significantly higher titers for all the adjuvanted groups compared to the non-adjuvanted control at day 21 (Supplemental Fig. 4a,b) and day 42 (Fig. 3a,b). In addition, when comparing the response from 21 to 42 days, there was an approximately tenfold increase in the IgG titers (Supplemental Fig. 4 and 5). At day 21, there were no significant differences in the titers between the three yeast-derived triterpene adjuvants compared to the shark squalene or the commercially available Addavax (Supplemental Fig. 4). The total IgG against the quadrivalent antigen results mirrored the individual IgG results; there were no significant differences between any of the yeast triterpenes and the Addavax or marine squalene controls, with significant differences only between the adjuvanted and non-adjuvanted groups (Fig. 3c). IgG1:IgG2a ratios were not statistically different when comparing the antigen only treatment to any of the adjuvanted vaccines. The mixed squalenes did give a slightly lower IgG1:IgG2a ratio and was significantly lower than shark squalene adjuvanted treatment (Fig. 4). IgE titers against the quadrivalent antigen were not detected above background in any of the treatment groups (data not shown) suggesting no allergic reactions triggered by any of the vaccines. In agreement with the ELISA data, the HI assay also showed significantly higher titers of HI antibodies in each of the adjuvant treatments compared to the antigen only treatment coinciding with the overall antibody responses (Fig. 5). We also assayed for the presence of IgE antibodies against botryococcene due to it being a non-native compound. No detectable levels of IgE antibodies against botryococcene above background were detected.

**Figure 6:**
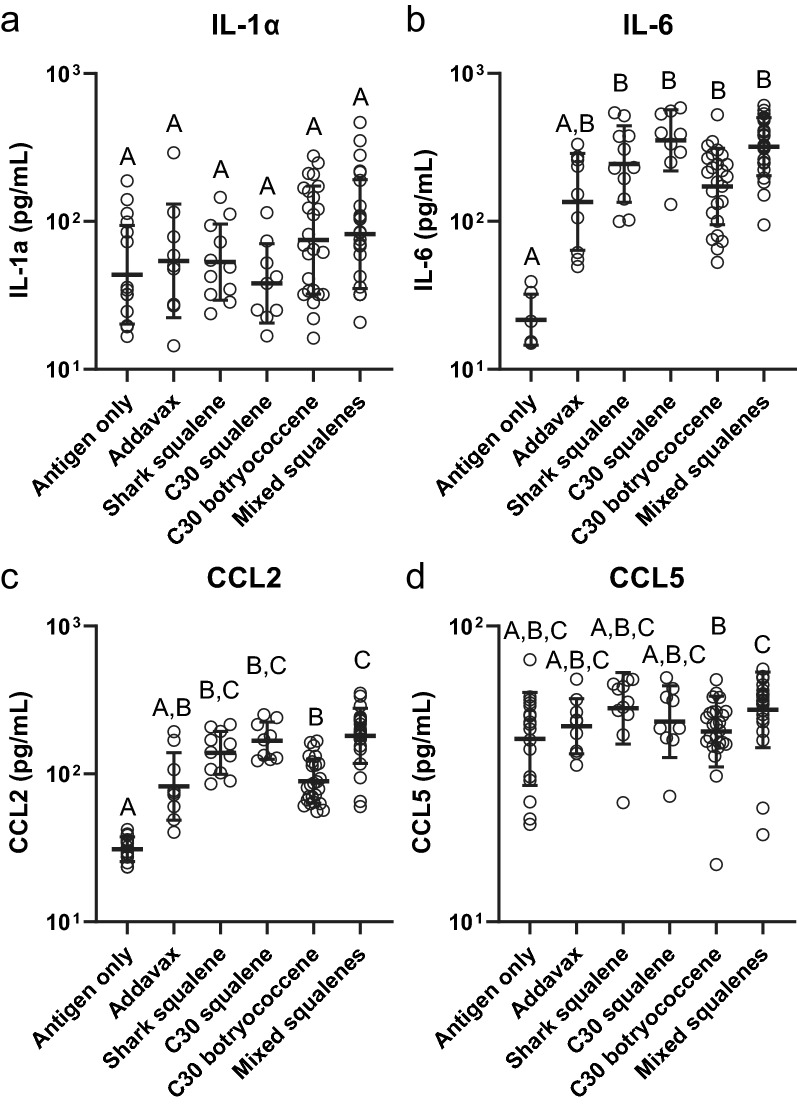
Immune inflammatory responses of mice to influenza vaccine formulated with different triterpene adjuvants. (**a**) IL-1α, (**b**) IL-6, (**c**) CCL2, and (**d**) CCL5 were the only detectable cytokines in serum using a multiplexed ELISA (14 cytokines assayed). Serum was taken 4 h after second injection (21 days after initial injection). Concentrations are presented from samples determined in duplicate (± geometric standard deviation). Group values were compared using a Kruskal–Wallis test with Dunn’s multiple comparison test (α = 0.05). Different letters above groups represent significant differences, shared letters represent no significant differences. Open circles represent individual animals. Antigen only (n = 17), Addavax (n = 9), shark squalene (n = 11), C30 squalene (n = 9), C30 botryococcene (n = 25), mixed squalenes (n = 25).

Sera collected four hours after the boost immunization (day 21) were analyzed for 14 cytokines in a multiplexed ELISA assay to determine potential differential inflammatory responses induced by the adjuvants. Only four cytokines were present at detectable levels in all treatments: IL-1α, IL-6, CCL2 and CCL5 (Supplementary Table 1). IL-1α, while detectable in all treatments, did not show any significant differences between administered vaccines (Fig. 6a). However, IL-6 (Fig. 6b) and CCL2 (Fig. 6c) both showed significantly elevated levels in all adjuvanted samples, except for Addavax (which did not show a significant difference from any of the treatments), when compared to the antigen-only control. Interestingly, the mixed squalene and C30 botryococcene adjuvanted treatments were also significantly different from each other in CCL2 (Fig. 6c). CCL5 levels exhibited no difference between non- and adjuvanted treatments, but again C30 botryococcene and mixed squalenes were significantly different from each other (Fig. 6d).

Comparing sex-based responses, females responded better to immunization than males, consistent with prior observations^38,39^. However, we did not observe statistical differences between the sexes in any of the treatments (Supplementary Fig. 4 and 5). This could be due to the reduced statistical power attributable to the smaller group sizes.

## Discussion

The key component of the MF59 adjuvant, squalene, harvested from shark liver oil is not a desirable source. In addition to ethical and sustainability issues, there is growing concern for the bioaccumulation and biomagnification of persistent organic pollutants (such as polybrominated diphenyl ethers) in shark-derived squalene^40^. This has caused all industries utilizing squalene to search for a renewable alternative. As previously discussed, plant-derived squalene has been put forward as a candidate. However, in addition to the difficulty in extraction and variation in purity, when olive-derived squalene was evaluated in an adjuvant formulation, it was found to be less stable than shark squalene—a difference that was attributed to lower purity of the olive squalene^41^. A microbial production platform has the benefit of a completely controlled, closed production system (which is important in current good manufacturing processes) and the ability to be scaled in proportion to demand (just-in-time manufacturing). This is especially relevant for use in a pandemic influenza adjuvant, where the time to produce vaccines to protect the population is critical to reduce the disease impact^42^. The ability to produce adjuvant quickly and easily would allow for dose-sparing of antigen (which often is a more laborious and time-intensive to produce), potentially resulting in a larger proportion of the populace being protected more quickly.

The results demonstrate the ability of Synthetic Biology-derived triterpene oils, of high purity and of non-animal origin, to perform statistically as well in an O/W adjuvant formulation when compared to the current source of squalene using standard immunological markers for this type of adjuvant/vaccine study (i.e. IgG1, IgG2a, total IgG, and HI titers)^23,43–45^. In both physiochemical and immunogenic properties, the Synthetic Biology produced squalene, botryococcene and methylated squalene performed equivalent to shark-derived squalene based on immunological activity. The stability of the Synthetic Biology adjuvants was also observed to persist over long periods of time with minimal change in the droplet size of the emulsions (Supplemental Fig. 3). We did not observe any reactogenic effects with botryococcene or methylated triterpene adjuvants, suggesting that although these oils are not normally found in the body (compared to squalene), they were well tolerated. These results bode well for further development for human vaccine applications.

There were no significant differences in the antibody titers between the biosynthetically derived squalene-based adjuvant compared to the shark-derived squalene or the commercially available Addavax for IgG1 or IgG2a. Nor was there a significant difference between the titers for the botryococcene and methylated squalene-based adjuvants which were found to perform equally well compared to shark squalene or Addavax (Fig. 3a,b). All the adjuvants had significantly higher titers at both the mid and final timepoint as compared to the non-adjuvanted control (Fig. 3, Supplemental Fig. 4 and 5). Total IgG against Afluria results also mirrored the individual IgG results: there were no significant differences between any of the yeast triterpenes versus the Addavax and marine squalene controls, with the only significant difference arising against the non-adjuvanted group (Fig. 3c). These results suggest that the origin of triterpene is unrelated to its effect, if sufficiently pure.

It is interesting to note that although the methylated squalene group produced similarly high antibody titers as compared to the other adjuvanted groups, it showed a difference in the IgG1: IgG2a ratio (Fig. 4). It has been previously reported that BALB/c mice respond to the influenza vaccine with a T helper type 2 (Th2) response, indicating a higher stimulation of IgG1, known to neutralize the viral particles^46–48^. However, the mixed squalene adjuvant had a lower IgG1:IgG2a ratio suggesting either a slightly lower or higher stimulation of IgG1 or IgG2a, respectively, as compared to the other groups. IgG2a antibody stimulation is equally important to the effectiveness of the influenza vaccine as it has been correlated with increased efficacy in its ability to clear the virus from the host^48^. While this difference in the IgG1:IgG2a ratio of the methylated squalene was subtle, it may suggest an additional layer to the efficacy of the adjuvanted influenza vaccine or a feature that might be important in future vaccine development.

In the cytokine analysis, there was a statistically significant difference when comparing the levels of CCL2 and CCL5 between botryococcene and the methylated squalene groups (Fig. 6c,d). Both CCL2 and CCL5 are involved in the recruitment of various leukocytes to the site of inflammation which can help stimulate components of adaptive immunity responsible for the efficacy of vaccination^49^. However, we cannot attribute any important physiological phenomena to the difference observed in the CCL2 and CCL5 levels in the methylated squalene and botryococcene adjuvants at this time. Although, this might support further investigation into mixtures of squalenes to test nuance responses in T cells and B cells with the goal of creating a more desirable immune response and a more tailored adjuvant.

In agreement with the ELISA data, the HI assay also showed much higher HI titers in each of the adjuvant treatments as compared to the antigen only treatment, mirroring the overall antibody responses (Fig. 5). The HI assay is widely accepted as directly corresponding to virus protection^50^, the current results are consistent with ability of Synthetic Biology-derived adjuvants to effectively substitute for shark-derived adjuvants. If these adjuvants are to be carried forward, future work examining true protection after viral challenge in a more appropriate animal model (e.g. ferrets)^51^ would be an important next step.

Through our findings, we have demonstrated the ability for yeast-derived triterpenes to work as adjuvants as well as shark squalene and commercially available Addavax. In addition to functional equivalency and efficacy of the biosynthetic squalene, we have also demonstrated the ability for botryococcene and methylated squalene—structurally distinct and novel triterpenes—to be functionally equivalent in their ability to elicit an immune response to influenza antigens. From the physical characteristics of the nanoemulsion to the antibody and cytokine responses, these Synthetic Biology-derived triterpenes performed equally well in comparison to the currently shark-sourced squalene. However, issues of purity, origin, and pollutant contamination makes these Synthetic Biology derived triterpenes potentially superior to the current conventional sources.

## Supplementary information

Supplementary Information.
