# Supplementary material for: Synthetic Biology-derived triterpenes as efficacious immunomodulating adjuvants
Source: Sci Rep. 2020 Oct 13;10:17090. doi: 10.1038/s41598-020-73868-6 (PMC7553918; doi:10.1038/s41598-020-73868-6)
Supplement: Supplementary file 1 — Supplementary Information. [file 41598_2020_73868_MOESM1_ESM.docx]

**SUPPLEMENTAL INFORMATION**

**Synthetic Biology-derived triterpenes as efficacious immunomodulating adjuvants**

Mizuki Tateno^1^, Barbara J. Stone^2^, Sarah J. Srodulski^2^, Stephanie Reedy^3^, Thomas R. Gawriluk^4^, Thomas M. Chambers^3^, Jerold Woodward^5^, Joe Chappell^1,4^, and Chase F. Kempinski^1,4^*


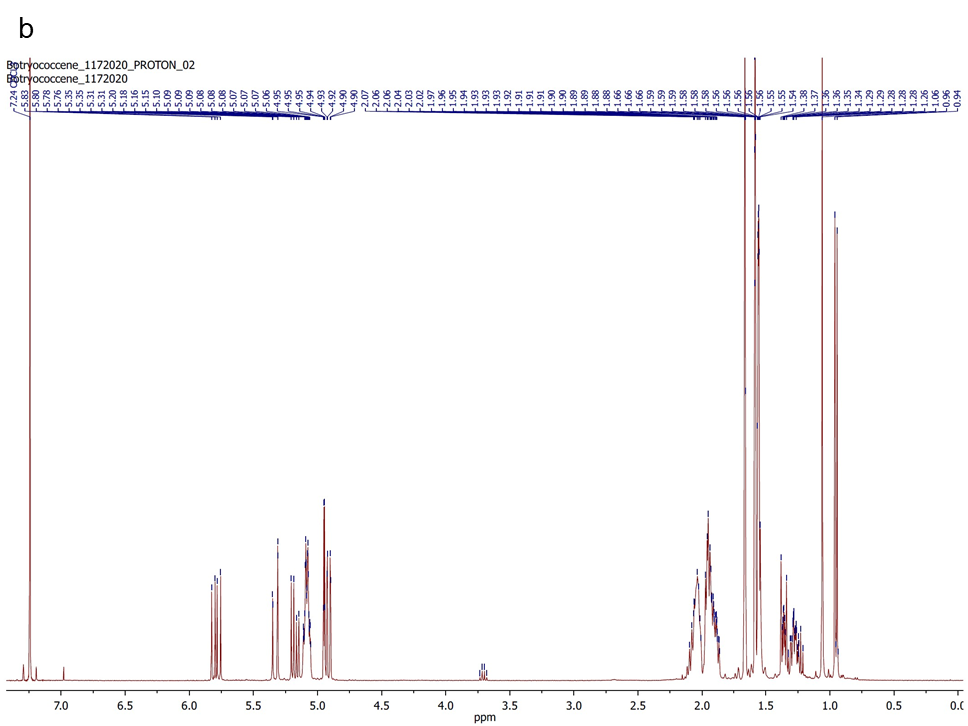

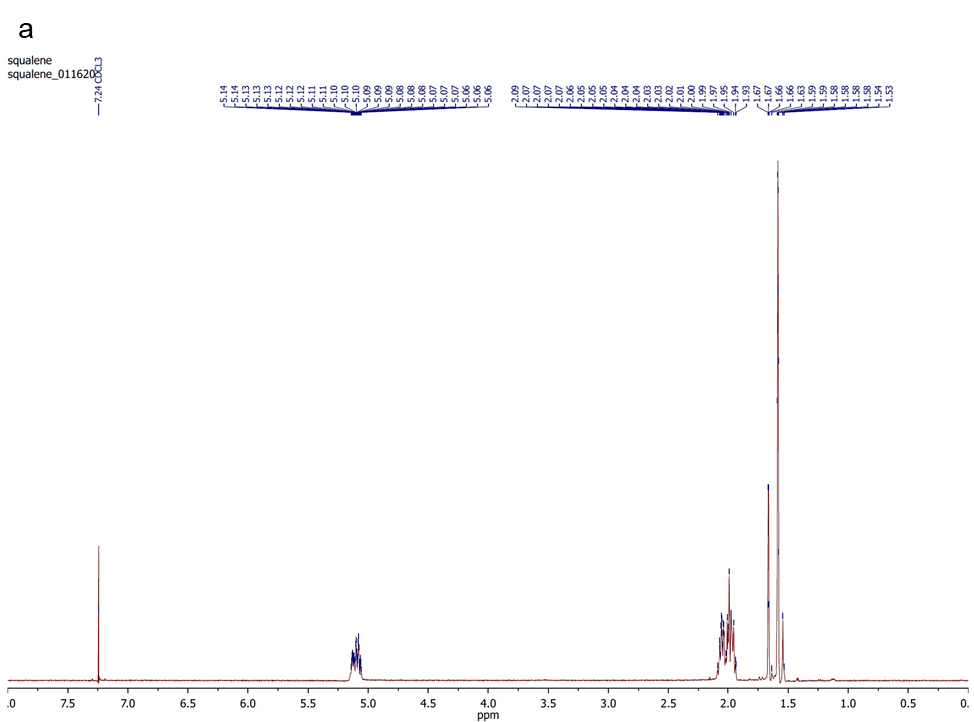
**Supplemental Figure 1.** (**a**) squalene and (**b**) botryococcene ^1^H-NMR of pure triterpene used in adjuvant formulations.


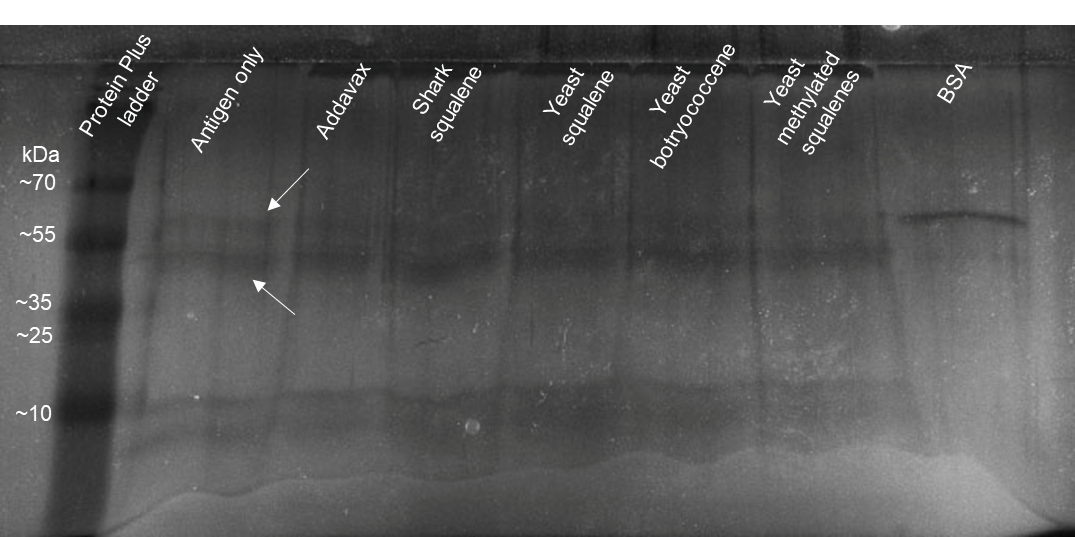


**Supplemental Figure 2.** Antigen integrity verified by SDS-PAGE and zinc staining. Arrows indicate the major bands in the antigen only lane, which most likely correspond to HA proteins that served as the antigens in the quadrivalent vaccine.


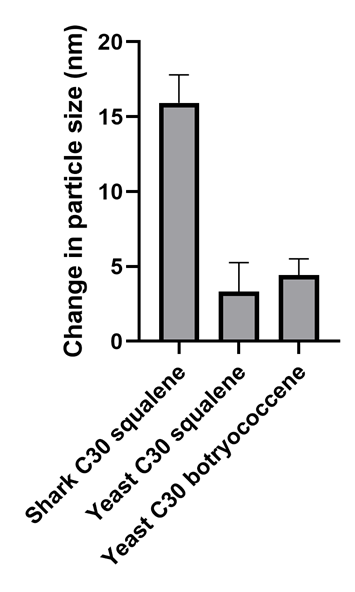


**Supplemental Figure 3.** Adjuvant particle size change after extended storage at 4^o^C.

**
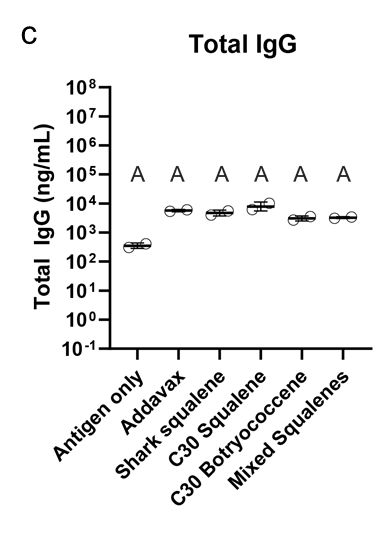

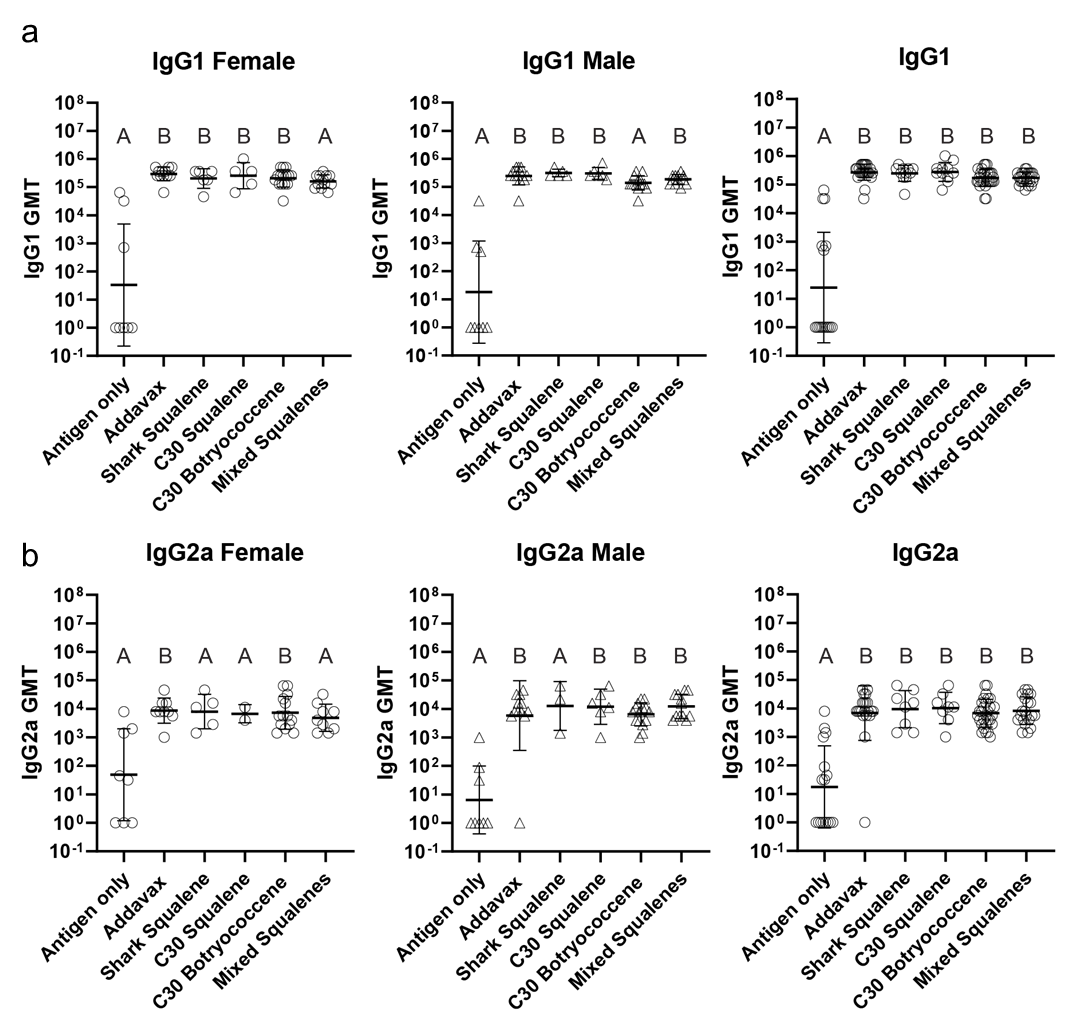
Supplemental Figure 4.** (**a**) IgG1, (**b**) IgG2A, and (**c**) Total IgG against quadrivalent antigen were quantified in serum using ELISA. Serum was taken 21 days after initial vaccination. Total IgG was analyzed by pooling male serum or female serum within each treatment (each data point represents pooled male or female sera). For IgG1: Antigen only male (n=8) female (n=8); Addavax male (n=12) female (n=12); C30 botryococcene male (n=15) female (n=15); Shark Squalene male (n=5) female (n=6); C30 Squalene male (n=6) female (n=5); Mixed squalenes male (n=12), female (n=12). For IgG2a: Antigen only male (n=8) female (n=8); Addavax male (n=12) female (n=9); C30 botryococcene male (n=15) female (n=14); Shark Squalene male (n=3) female (n=5); C30 Squalene male (n=6) female (n=2); Mixed squalenes male (n=12), female (n=9). Group values were compared using a Kruskal-Wallis test with Dunn’s multiple comparison test (α=0.05). Different letters above groups represent significant differences, shared letters represent no significant difference.

**
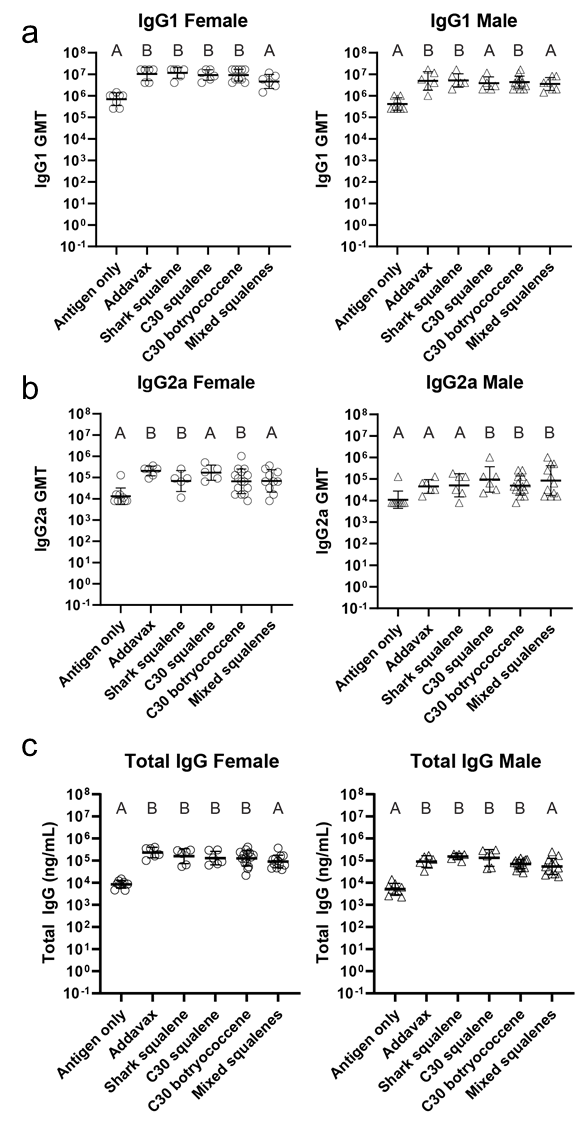
Supplemental Figure 5.** Male and female (**a**) IgG1, (**b**) IgG2a, and (**c**) Total IgG against quadrivalent antigen were quantified in serum using ELISA. Serum was taken on day 42. In IgG1: Addavax male (n=6) female (n=6); Antigen only male (n=8) female (n=8); Shark Squalene male (n=6) female (n=6); C30 Squalene male (n=6) female (n=6); C30 botryococcene male (n=13) female (n=10); Mixed squalenes male (n=7), female (n=6). For IgG2a: Addavax male (n=6) female (n=6); Antigen only male (n=9) female (n=9); Shark Squalene male (n=6) female (n=5); C30 Squalene male (n=6) female (n=5); C30 botryococcene male (n=14) female (n=15); Mixed squalenes male (n=11), female (n=10). For total IgG: Addavax male (n=6) female (n=6); Antigen only male (n=9) female (n=9); Shark Squalene male (n=6) female (n=6); C30 Squalene male (n=6) female (n=6); C30 botryococcene male (n=15) female (n=15); Mixed squalenes male (n=12), female (n=12). Group values were compared using a Kruskal-Wallis test with Dunn’s multiple comparison test (α=0.05). Different letters above groups represent significant differences, shared letters represent no significant differences.

**Supplemental Table 1.** Cytokines assayed in multiplex from sera taken 4 hours after the boost immunization on day 21. Only those highlighted exhibited detectable levels. Due to limitations on sera volumes, the number of animals assayed is indicated below each treatment.

| **Cytokine** | **Associated response** | **Antigen only (n=11)** | **Addavax**  **(n=17)** | **C30 botryococcene (n=25)** | **Shark squalene (n=11)** | **C30 Squalene (n=9)** | **Mixed squalenes (n=25)** |
| --- | --- | --- | --- | --- | --- | --- | --- |
| IL-1α | Th2 | Response | Response | Response | Response | Response | Response |
| IL-1β | Th1 | Not Present | Not Present | Not Present | Not Present | Not Present | Not Present |
| IL-2 | Th1 | Not Present | Not Present | Not Present | Not Present | Not Present | Not Present |
| IL-3 |  | Not Present | Not Present | Not Present | Not Present | Not Present | Not Present |
| IL-4 | Th2 | Not Present | Not Present | Not Present | Not Present | Not Present | Not Present |
| IL-6 | Th2 | Response | Response | Response | Response | Response | Response |
| IL-10 | Th2 | Not Present | Not Present | Not Present | Not Present | Not Present | Not Present |
| IL-12 | Th1 | Not Present | Not Present | Not Present | Not Present | Not Present | Not Present |
| IL-17 | Th1 | Not Present | Not Present | Not Present | Not Present | Not Present | Not Present |
| CCL2 | Th1 | Response | Response | Response | Response | Response | Response |
| TNFa | Th1 | Not Present | Not Present | Not Present | Not Present | Not Present | Not Present |
| CCL3 | Th1 | Not Present | Not Present | Not Present | Not Present | Not Present | Not Present |
| CSF2 |  | Not Present | Not Present | Not Present | Not Present | Not Present | Not Present |
| CCL5 | Th1 | Response | Response | Response | Response | Response | Response |
